# Effect of hormone-induced plasma membrane phosphatidylinositol 4,5-bisphosphate depletion on receptor endocytosis suggests the importance of local regulation in phosphoinositide signaling

**DOI:** 10.1038/s41598-023-50732-x

**Published:** 2024-01-02

**Authors:** Dániel J. Tóth, József T. Tóth, Amir Damouni, László Hunyady, Péter Várnai

**Affiliations:** 1 Department of Physiology, Faculty of Medicine, Semmelweis University, Budapest, Tűzoltó utca 37-47, 1094 Hungary; 2 HUN-REN–SU Molecular Physiology Research Group, Hungarian Research Network and Semmelweis University, Budapest, Hungary; 3 Department of Anaesthesiology and Intensive Therapy, Faculty of Medicine, Semmelweis University, Budapest, Üllői út 78/B, 1082 Hungary; 4 Institute of Enzymology, Centre of Excellence of the Hungarian Academy of Sciences, HUN-REN Research Centre for Natural Sciences, Budapest, Magyar tudósok körútja 2, 1117 Hungary

**Keywords:** Phosphoinositol signalling, Sensors and probes

## Abstract

Phosphatidylinositol 4,5-bisphosphate (PIP2) has been shown to be critical for the endocytosis of G protein-coupled receptors (GPCRs). We have previously demonstrated that depletion of PIP2 by chemically induced plasma membrane (PM) recruitment of a 5-phosphatase domain prevents the internalization of the β2 adrenergic receptor (β2AR) from the PM to early endosomes. In this study, we tested the effect of hormone-induced PM PIP2 depletion on β2AR internalization using type-1 angiotensin receptor (AT1R) or M3 muscarinic acetylcholine receptor (M3R). We followed the endocytic route of β2ARs in HEK 293T cells using bioluminescence resonance energy transfer between the receptor and endosome marker Rab5. To compare the effect of lipid depletion by different means, we created and tested an AT1R fusion protein that is capable of both recruitment-based and hormone-induced depletion methods. The rate of PM PIP2 depletion was measured using a biosensor based on the PH domain of phospholipase Cδ1. As expected, β2AR internalization was inhibited when PIP2 depletion was evoked by recruiting 5-phosphatase to PM-anchored AT1R. A similar inhibition occurred when wild-type AT1R was activated by adding angiotensin II. However, stimulation of the desensitization/internalization-impaired mutant AT1R (TSTS/4A) caused very little inhibition of β2AR internalization, despite the higher rate of measurable PIP2 depletion. Interestingly, inhibition of PIP2 resynthesis with the selective PI4KA inhibitor GSK-A1 had little effect on the change in PH-domain-measured PM PIP2 levels but did significantly decrease β2AR internalization upon either AT1R or M3R activation, indicating the importance of a locally synthetized phosphoinositide pool in the regulation of this process.

## Introduction

Phosphoinositides are low-abundance phospholipids that are present in all kinds of biological membranes in eukaryotic cells. Although they were originally discovered as substrates for signaling molecules such as inositol 1,4,5-trisphosphate [Ins(1,4,5)P3] and phosphatidylinositol 3,4,5-trisphosphate (PIP3), intensive research over the past decades has led to the elucidation of their many other functions. There are phosphoinositides that provide energy for transport processes between membranes, but their most general function is to identify the membrane of various cell organelles. Their appearance in membranes both affects the function of specific membrane proteins and, based on their interactions with cytoplasmic proteins, leads to the formation of membrane-specific complexes or initiation of membrane-specific signaling processes^1^. Their membrane level is highly dynamic since it is controlled by various kinases, phosphatases, lipases and several newly discovered transporters^2,3^.

Phosphatidylinositol 4,5-bisphosphate (PIP2), the most investigated phosphoinositide, is the hallmark of the inner layer of the plasma membrane (PM). Although PIP2 is mainly produced by the phosphorylation of PM phosphatidylinositol 4-phosphate (PI4P) by type I phosphatidylinositol-phosphate kinases (PIPKs), alternative routes, such as the phosphorylation of phosphatidylinositol 5-phosphate by type II PIPKs or dephosphorylation of PIP3 by PTEN, cannot be excluded. The level of PM PIP2 can be decreased by phospholipase C (PLC) upon activation of several plasma membrane receptors, such as G protein-coupled receptors (GPCRs), or by phosphatidylinositol 3-kinase (PI3K) upon activation of receptor tyrosine kinases (RTKs). These enzymes use PIP2 as a substrate to generate second messengers, but PIP2 by itself can work as a signaling molecule to recruit molecules to the PM or by binding to PM proteins and provide them with information about membrane identity. There are molecular mechanisms that make PM PIP2 an essential determinant of plasma membrane functions, such as vesicular transport, ion channel regulation and actin organization^4,5^. How can PIP2 serve so many different functions? Several studies indicate the existence of different plasma membrane PIP2 pools and suggest that the regulatory roles of these distinct lipid pools may vary^6,7^, but it has not been properly assessed whether, for example, the absolute level of PM PIP2 or its turnover are more important^8,9^.

In the present study, we used a Gq-coupled receptor specifically designed to investigate the consequence of PIP2 depletion created at the same location within the PM but with different mechanisms. We found that the same decrease in the steady-state PIP2 level was inhibitory for the endocytic machinery of a Gs-coupled receptor if lipid depletion was achieved by the recruitment of a 5-phosphatase enzyme to the receptor but not when PLC-dependent PIP2 hydrolysis was initiated by direct activation of the receptor. Surprisingly, in the presence of GST-A1 (A1), which selectively inhibits type IIIα phosphatidylinositol 4-kinase (PI4KA), the kinase responsible for the resynthesis of PM PI4P and PIP2, PLC activation was also able to inhibit the endocytic process, suggesting the importance of local de novo synthesis of PIP2.

## Results

### Development of a molecular tool capable of both agonist- and chemically induced PM PIP2 depletion

**Figure 1:**
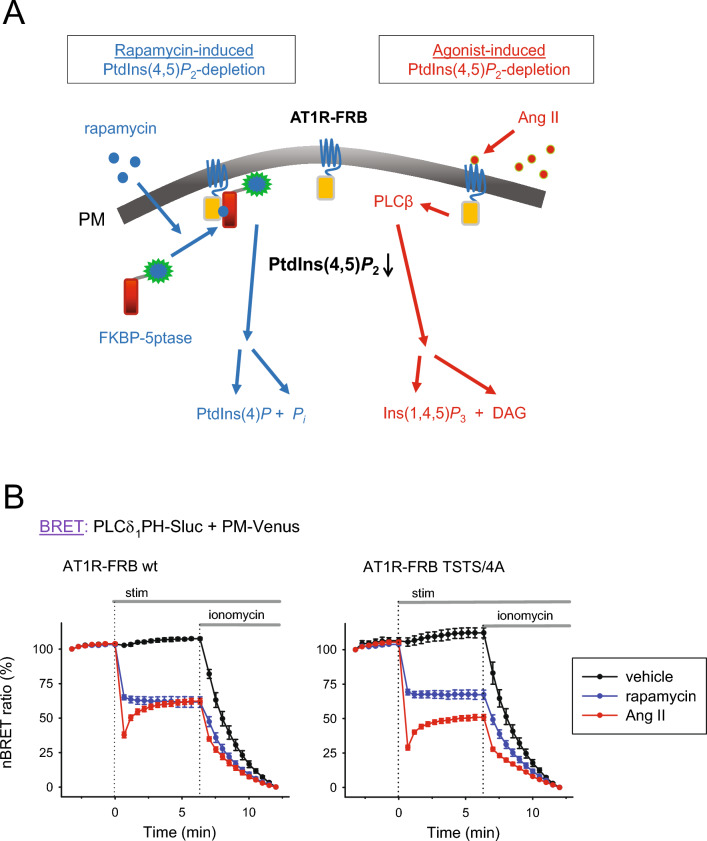
Two different ways to deplete PM PIP2 within one system. (**A**) Schematic depiction of the system for PIP2 depletion via two different pathways. It consists of AT1R-FRB and FKBP-5-phosphatase (FKBP-5ptase), both tagged with mRFP (not shown here). Adding rapamycin induces the heterodimerization of FRB and FKBP, which recruits FKBP-5ptase to the plasma membrane, where it then dephosphorylates PIP2 (blue arrows). In contrast, stimulation of AT1R-FRB with Ang II activates PLCβ, which cleaves the phospholipid into Ins(1,4,5)P3 and DAG (red arrows). Abbreviations: Pi: inorganic phosphate, PLCβ: phospholipase Cβ, DAG: diacylglycerol. (**B**) HEK 293T cells were transfected with the L10-Venus-T2A-PLCδ1PH-Sluc biosensor, allowing the measurement of PM PIP2 depletion efficiency by calculating BRET between PM-targeted Venus (PM-Venus) and the luciferase-tagged PH domain of PLCδ1 (PLCδ1PH-Sluc), which binds to PIP2 specifically. The cells also expressed FKBP-5ptase and AT1R-FRB (either wild type (wt)—on the left—or the desensitization and internalization deficient mutant, TSTS/4A—on the right), both tagged with mRFP. Lipid depletion was triggered by either AT1R stimulation (100 nM Ang II, red curves) or rapamycin addition (300 nM, blue curves) at time point 0, followed by ionomycin (10 μM) for complete degradation of PIP2. The signal change was normalized to the first and last data points (100% and 0%, respectively). Black curves show unstimulated samples (only ionomycin was given). Data show the mean ± SEM of 5 independent experiments, each performed in triplicate.

To test whether the consequences of PIP2 depletion depend on the mechanism by which lipid depletion is induced, we created a type-1 angiotensin receptor (AT1R) tagged with the FKBP and rapamycin-binding (FRB) domain of mTOR on its C-terminal end. The advantage of this protein construct is that when coexpressed with a previously used FK506-binding protein (FKBP)-tagged 5-phosphatase (5ptase) enzyme^10^, it allows PM PIP2 depletion by adding either angiotensin II (Ang II) or rapamycin under the same conditions; therefore, the effect of the two different types of PIP2 depletion (agonist-induced or “physiological” and chemically induced or “artificial”) can be compared. As shown in Fig. 1A, in cells expressing AT1R-FRB, the addition of Ang II causes agonist-induced activation of the receptor, which leads to PLCβ activation and thus Ins(1,4,5)P3 and diacylglycerol (DAG) production, while the addition of rapamycin induces heterodimerization between FRB and FKBP, therefore recruiting the 5ptase domain to the PM, where it catalyzes the PIP2 to PI4P conversion.

To compare the effectiveness of agonist- and chemically induced PIP2 depletion, we used our previously described bioluminescence resonance energy transfer (BRET)-based PM PIP2 biosensor^11,12^. This single-plasmid biosensor contains the Venus fluorescent protein bound to a short plasma membrane target sequence (L10—the first ten amino acid residues of the Lck protein) and the PIP2-sensitive PLCδ_1_-PH domain^13^ fused to *Renilla* luciferase enzyme. These two sequences are connected in the plasmid by the “self-cleaving” viral T2A peptide coding sequence, leading to the expression of two separate proteins in equimolar amounts^14^. We transiently transfected HEK 293T cells with the AT1R-FRB and FKBP-5ptase constructs and the plasmid of the PIP2 biosensor and stimulated the cells with either 100 nM Ang II or 300 nM rapamycin. As shown in Fig. 1B, PM PIP2 levels decreased rapidly when either an AT1R agonist was added (red traces) or, with rapamycin, the 5ptase enzyme was recruited to AT1R (blue traces). The addition of the Ca^2+^ ionophore ionomycin (10 μM), which leads to the activation of calcium-dependent PLCs, could further increase the rate of PIP2 depletion, indicating that neither the hormone- nor the chemically induced lipid depletion system could evoke a complete elimination of the PM PIP2 pool. Although the addition of Ang II initially resulted in greater depletion compared to rapamycin, it was transient, and after several minutes, the effectiveness of the depletion became indistinguishable when we used the wild-type AT1R construct (left panel).

Expressing the desensitization and internalization-impaired AT1R (TSTS/4A^15,16^.) instead of the wild type did not influence the effect of rapamycin on PIP2 depletion but was more robust upon Ang II stimulation (right panel), indicating that desensitization of wild-type AT1R-FRB limits its capacity for agonist-induced PIP2 depletion. Overall, it appears that the AT1R-FRB constructs are capable of inducing PIP2 depletion by different mechanisms in the same cellular system, depending on whether Ang II or rapamycin is added.

### Comparison of the effect of PM PIP2 depletion by agonist-induced PLC activation and chemically induced 5ptase PM recruitment on β2AR endocytosis

Phosphoinositides play important roles in receptor endocytosis, and artificially influencing their levels can inhibit several steps in this complex and strictly regulated process^17–20^. To investigate in deeper detail the role of PM PIP2, particularly the mechanism of PIP2 depletion, we decided to examine how Ang II- or rapamycin-induced PIP2 depletion affects the endocytosis (internalization) of the β2 adrenergic receptor (β2AR). To do this, we applied our previously developed BRET-based system, in which luciferase-labeled receptors and Venus-tagged Rab5, as a marker for early endosomes, were used. In this system, arrival of the internalized β2AR-luc at the early endosomal compartment was indicated by a rise in the BRET between the receptor and Venus-Rab5^21–23^. Internalization of β2AR was initiated by the addition of 1 μM isoprenaline to HEK 293T cells.

**Figure 2:**
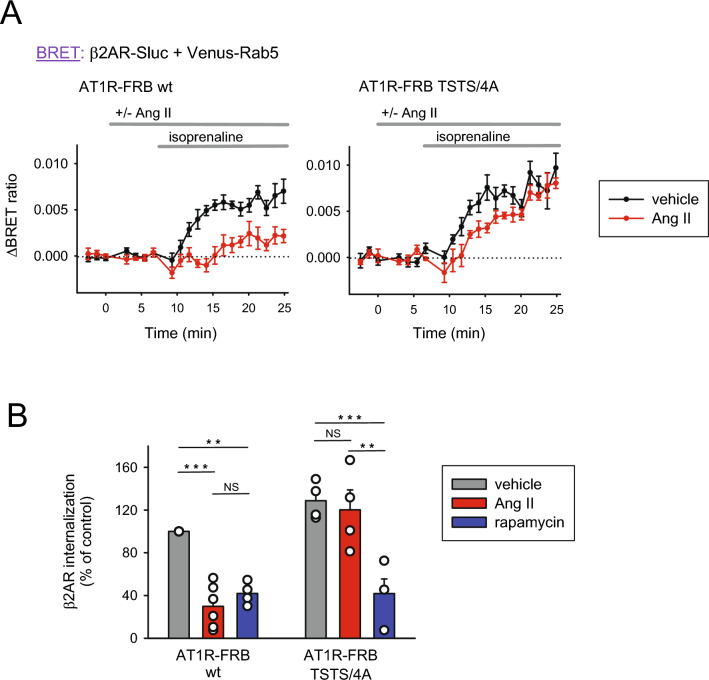
Effect of hormone-induced PM PIP2 depletion on β2AR endocytosis in HEK 293T cells. (**A**) Internalization of β2AR was measured by BRET between β2AR-Sluc and Venus-Rab5 in HEK 293T cells also expressing AT1R-FRB (wt or TSTS/4A mutant, left and right, respectively) and FKBP-5ptase, again, both mRFP-tagged. Cells were treated with vehicle (black curves) or Ang II (100 nM, red curves) for approximately 5 min before β2AR-Sluc was stimulated with isoprenaline (1 μM). The BRET ratio change was calculated against nonstimulated baselines (not shown) for each curve. Data are the mean ± SEM of 4–6 independent experiments, each performed in triplicate. (**B**) The bar graph shows the rate of β2AR-Sluc internalization 10–15 min after isoprenaline (1 μM) treatment normalized to the wt AT1R-FRB vehicle-treated sample (gray bar on the left). Data are means (± SEM) of the last 5 measurement points of the curves from panel A, complemented with data from cells treated with rapamycin (300 nM, green bars) instead of Ang II in the same experiment. Data were analyzed using two-way ANOVA, which showed a significant difference (*p* < 0.05) between the groups concerning both the type of AT1R-FRB used and the treatments, and there was also a significant interaction between the two aspects. The Bonferroni's post hoc test was used for pairwise comparisons within groups, and the marked pairs were found to be significantly different. **p < 0.01; ***p < 0.001; n = 3–6.

As shown in Fig. 2A, after proper optimization of the DNA amounts used for transfection, agonist-induced β2AR internalization was clearly detectable when cells were stimulated with isoprenaline (black traces). Interestingly, the signal was even greater when the mutant AT1R was expressed in the cells. However, the real surprise was the consequence of agonist-induced PIP2 depletion. As shown in Fig. 1B, the addition of Ang II caused a larger PIP2 depletion in the case of the nondesensitizing mutant receptor; therefore, a stronger inhibitory effect on internalization was expected. However, we experienced the opposite. Initiation of β2AR internalization by isoprenaline after 7 min of Ang II addition resulted in unambiguous inhibition of β2AR internalization in cells expressing wild-type AT1R, while it was basically unchanged in the case of internalization-impaired, mutant AT1R (red traces). This difference is more conspicuous on a bar graph (Fig. 2B), which contains the statistical analysis of these experiments. The bars show β2AR internalization as the average of the BRET ratio changes at the last 5 measurement points (10–15 min after isoprenaline was added). In line with our previous results^23^, using 300 nM rapamycin pretreatment instead of Ang II caused a significant and comparable inhibitory effect on the endocytosis of β2AR regardless of which AT1R-FRB construct was transfected into the cells (blue bars).

Although in the case of the desensitization-impaired AT1R-FRB and 5ptase-FKBP protein pair we found that the regulation of a PIP2-dependent process depended on the mechanism by which PM PIP2 was depleted, we also wanted to understand what was behind the difference between wild-type AT1R-FRB and AT1R-TSTS/4A-FRB. A possible explanation is that stimulated wild-type AT1R-FRB also undergoes endocytosis and may use the same endocytic machinery as β2AR, and the two receptors might compete for endocytic partner molecules. To test this hypothesis, we performed the same series of experiments as shown in Fig. 2, but in addition to the previously used constructs, we transfected the cells with β-arrestin 2 to enhance a possible rate-limiting step in the endocytic process. As shown in Supplementary Fig. S1, in the case of the mutant AT1R, overexpression of β-arrestin 2 essentially did not influence the effect of Ang II-induced PIP2 depletion on β2AR internalization; at most, the transient delay became more prominent but after 8–10 min, the amount of receptors that arrived at the early endosome was the same as that without PIP2 depletion. In contrast, in the case of wild-type AT1R, the addition of Ang II and the consequential receptor activation and PIP2 depletion also did not cause the inhibition of β2AR internalization. These results are compatible with the hypothesis that activated receptors compete for the components of the endocytic machinery when activated in parallel. Therefore, in further experiments, we used noninternalizing receptors such as the TSTS/4A mutant of AT1R or the type-3 muscarinic acetylcholine receptor (M3R) (see later).

### Investigating the role of subsequent local PIP2 resynthesis after AT1 receptor activation in the endocytosis of β2AR

As shown in Fig. 1B, both the agonist- and rapamycin-induced mechanisms resulted in comparable PIP2 depletion regardless of the receptor used. It is important to note that in our assay, we measured the steady-state level of PIP2, which can be achieved by having very different turnover rates of PIP2. Although early reports of PLCδ1-PH-based PIP2 measurements indicated that this PH domain can interact and “see” the hormone-sensitive PIP2 pool in the PM^13^, the crosstalk between the various pools and even the exact identification of these proposed PIP2 pools is still not known. Through studies on the synthesis of PIP2 in the PM, it has been established that type I PIPKs can quickly phosphorylate PI4P at the PM, and thus, the rate-limiting step of PIP2 synthesis is PI4P genesis via PI4Ks^24^. It is also known that PM PIP2 levels can be maintained during agonist stimulation as long as the PI4KA enzyme is able to synthesize PI4P at the PM^25^, and some of our previous data suggest that PIP2 resynthesis is initiated not by the decrease in PI4P but rather by activation of the Ins(1,4,5)P3—DAG pathway^12^. Thus, when stimulating AT1R-FRB (TSTS/4A) with Ang II, there is a chance that consequential PI4KA activation will lead to PI4P production sufficient to quickly replenish the hormone-sensitive PIP2 pool. If this pool is responsible for receptor-mediated endocytosis, replenishment via PI4KA allows β2AR internalization even when the total PIP2 content in the PM is not entirely restored. To test this hypothesis, we again investigated β2AR internalization after activating AT1R under conditions where PI4P resynthesis upon PLC activation was blocked. To achieve this, we used GSK-A1 (hereafter referred to as A1), a specific PI4KA inhibitor^26^.

**Figure 3:**
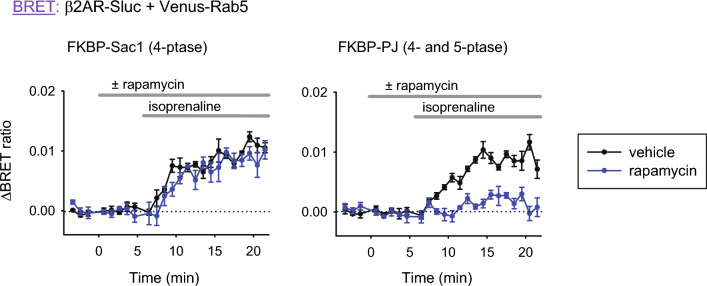
Effect of PM PI4P depletion on β2AR internalization. HEK 293T cells were transfected with β2AR-Sluc and Venus-Rab5 for monitoring receptor internalization as detailed before, along with PM-targeted FRB and FKBP-Sac1, a PI4P (left panel) or FKBP-Pseudojanin (FKBP-PJ) that has both 4- and 5-phosphatase activity (right panel), for rapamycin-induced depletion of PI4P only or PI4P and PIP2 combined, respectively. (For a detailed description of these constructs, see^27^). Cells were treated with rapamycin (300 nM, blue curves) or vehicle (black curves) and after 5 min stimulated with isoprenaline (1 μM). The BRET ratio change was calculated by subtracting baselines not stimulated with isoprenaline. Data show the mean ± SEM of 3 independent experiments, each performed in triplicate.

Since the addition of A1 depletes PM PI4P, we first checked whether this change has any effects on β2AR internalization. To do this, we again used our rapamycin-induced heterodimerization system, but this time we used the L10 sequence to target Venus to the PM and replaced the FKBP-5ptase construct with FKBP-Sac1 (4-phosphatase), which is able to dephosphorylate PI4P, or with FKBP-Pseudojanin, which has both 4- and 5-phosphatase activity^27^. Their effects on PM PI4P and PIP2 levels were demonstrated earlier^12^. Figure 3 shows that when the PM PI4P level was selectively lowered, it had no effect on the endocytosis of β2AR (left panel, blue curve), but when both lipids were eliminated from the PM, β2AR endocytosis did not occur (right panel, blue curve). These results indicate that initiation of the machinery of receptor-mediated endocytosis can tolerate the decreased PM PI4P level, and A1 can be used for further experiments.

**Figure 4:**
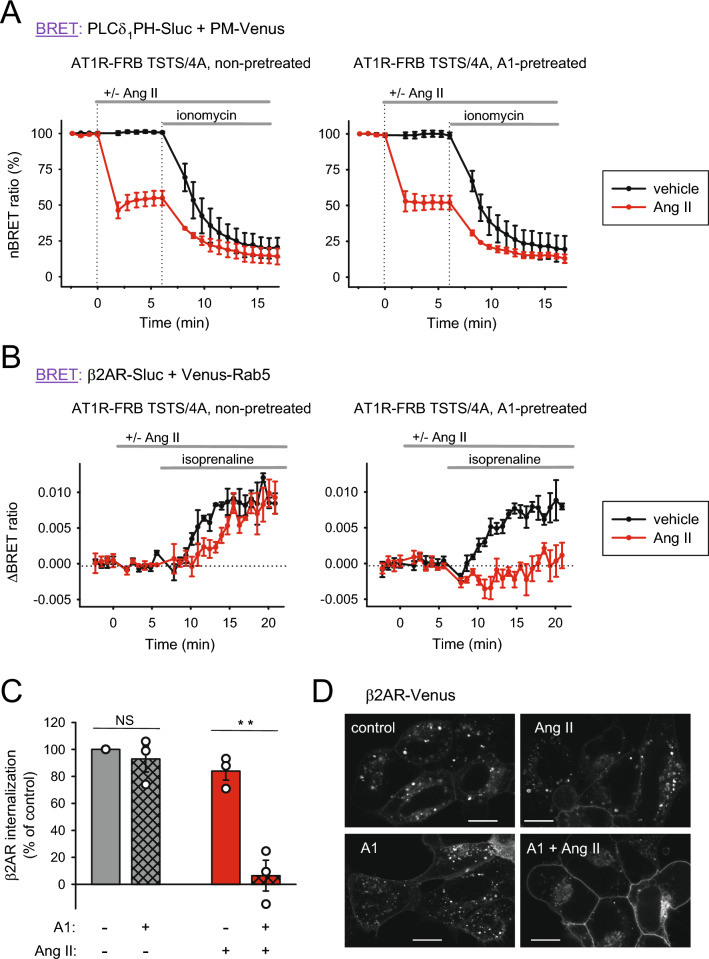
Importance of PI4P resynthesis by PI4KA on agonist-induced PIP2 depletion and receptor internalization. (**A**) PIP2 depletion efficiency was measured by BRET as detailed earlier in Fig. 1B, using the TSTS/4A mutant AT1R-FRB and applying a pretreatment for 10 min with either vehicle (left graph) or 10 nM GSK-A1 (A1), a specific inhibitor of PI4KA (right graph). Lipid depletion was triggered by AT1R stimulation (100 nM Ang II, red curves) at time point 0, followed by ionomycin (10 μM) for complete degradation of PIP2. The signal change was normalized to the first data point (100%) and the value of Renilla luciferase alone (0%). Black curves show unstimulated samples. Data show the mean ± SEM of 3 independent experiments, each performed in triplicate. (**B**) Internalization of β2AR was measured by BRET as detailed in Fig. 2A, with the distinction that the TSTS/4A mutant AT1R-FRB was expressed in all cells. Cells were pretreated with either vehicle (left panel) or A1 (10 nM, right panel) for 10 min. PIP2 depletion was evoked by activating AT1R-FRB at time point 0 with Ang II (100 nM, red curves). Black curves show data where cells were not treated with Ang II. After 5 min, β2AR-Sluc was stimulated with isoprenaline (1 μM). BRET ratio change was calculated by subtracting baselines not stimulated with isoprenaline. Data show the mean ± SEM of 3 independent experiments, each performed in triplicate. (**C**) The bar graph shows the rate of β2AR-Sluc internalization 10–15 min after isoprenaline (1 μM) treatment normalized to that in the nonpretreated and non-PIP2-depleted samples (clear gray bar). Data are the means (± SEM) of the last 5 measurement points of the curves from panel B. Data were analyzed using two-way ANOVA, which showed a significant difference (*p* < 0.01) between the groups and a significant interaction between Ang II and A1 treatments (*p* < 0.05). Bonferroni's post hoc test was used for pairwise comparisons, and only the A1-pretreated Ang II-stimulated sample was found to be significantly different from all others (*p* < 0.01). n = 3. (**D**) Internalization of β2AR was visualized by confocal microscopy. β2AR-Venus and AT1R TSTS/4A-mRFP were expressed in HEK 293A cells pretreated with vehicle (upper images) or A1 (10 nM, 10 min, lower images). PIP2 depletion was induced by activating AT1R with Ang II (100 nM, 5 min, right images only), and then β2AR was stimulated in all cells with isoprenaline (1 μM) for 15 min. Cells were then fixed, and confocal images of β2AR-Venus were taken. Representative images from three independent experiments. Scale bar: 15 μm.

First, as shown in Fig. 4A, HEK 293T cells expressing AT1R-TSTS/4A-FRB, FKBP-5ptase and the PIP2 biosensor were stimulated either with vehicle (black traces) or 100 nM Ang II (red traces) under control conditions (left panel) and after pretreatment with 10 nM A1 for 10 min (right panel). Since A1 pretreatment did not cause a significant change in the resting PM PIP2 levels, values were calculated as a percent of basal levels. A1 pretreatment also did not influence the extent of PIP2 depletion measured after 5 min of hormonal stimulation (red traces).

Next, we transfected HEK 293T cells with AT1R-TSTS/4A-FRB, β2AR-luc and Venus-Rab5 constructs and followed the endocytosis of β2AR after PLCβ activation by Ang II in control cells and in cells pretreated with 10 nM A1 for 10 min (Fig. 4B). The traces shown in the left panel of Fig. 4B are identical to the traces of Fig. 2A right panel but show the averages of another series of experiments. As expected, A1 pretreatment itself did not affect the endocytosis of β2AR to early endosomes (compare the black traces of the left and right panels). As shown before, in control cells (without A1 pretreatment), PLCβ activation via AT1R-TSTS/4A-FRB stimulation and the subsequent measurable PM PIP2 depletion did not alter the endocytosis of β2AR (red trace, Fig. 4B left panel) but significantly reduced it when PI4KA was inhibited (red trace, Fig. 4B right panel). Figure 4C again presents the average BRET ratio values of the last 5 measurement points from the experiments shown in Fig. 4B on a bar graph. Statistical analysis revealed that neither A1 pretreatment alone nor depletion of the PM PIP2 pool via PLCβ activation caused a significant decrease in the endocytosis of β2AR. In contrast, when the resynthesis of PI4P was inhibited with A1 prior to the hormonal stimulation of AT1R-TSTS/4A-FRB, β2AR internalization diminished significantly.

To visually confirm our findings on β2AR internalization, we performed confocal microscopy measurements in an experimental setup similar to that shown in Fig. 4B, with a few distinctions. HEK 293A cells were used because they are more suitable for confocal imaging, and Venus-tagged β2AR was used for visualizing internalization without the need for an endosomal marker. β2AR-Venus was transiently expressed in the cells along with AT1R-TSTS/4A-mRFP, and after 10 min of vehicle or A1 (10 nM) pretreatment, PIP2 depletion was induced in some samples by adding 100 nM Ang II for 5 min. β2AR was then stimulated by isoprenaline (1 µM) and allowed to internalize for 15 min, when cells were fixed and prepared for imaging. As shown in the representative images in Fig. 4D, in control cells pretreated with vehicle and with no lipid depletion, β2AR-Venus appeared predominantly in intracellular vesicles 15 min after stimulation (upper left image), indicating endocytosis of the receptor. PIP2 depletion or A1 treatment alone led to a similar picture with several intracellular puncta in each cell (upper right and lower left images, respectively). However, combining A1 pretreatment with Ang II-induced PIP2 depletion largely prevented receptor internalization; β2AR-Venus stayed on the PM with very few intracellular puncta (lower right image). This aligns well with our results from BRET experiments, and taken together, these data suggest that although endocytosis is a PIP2-sensitive process, it can occur even if the entire PIP2 pool of the PM is reduced by PLCβ as long as the PI4KA enzyme is able to replenish the hormone-sensitive PIP2 pool.

### Investigating the importance of PM PIP2 resynthesis for β2AR endocytosis upon different levels of M3R activation

To further investigate the importance of a local PIP2 pool generated by PI4KA on the endocytosis of β2AR, we aimed to perform a similar set of experiments using cells expressing another G_q_-coupled receptor. We chose M3R (type-3 muscarinic receptor) because it can evoke robust PLCβ activation and reportedly shows very slow desensitization^28^; thus, competition for the endocytic machinery between M3R and β2AR will not influence the results. In this series of experiments, the AT1R-TSTS/4A-FRB construct was replaced with M3R, and FKBP-5ptase was omitted, but all other circumstances were unchanged.

**Figure 5:**
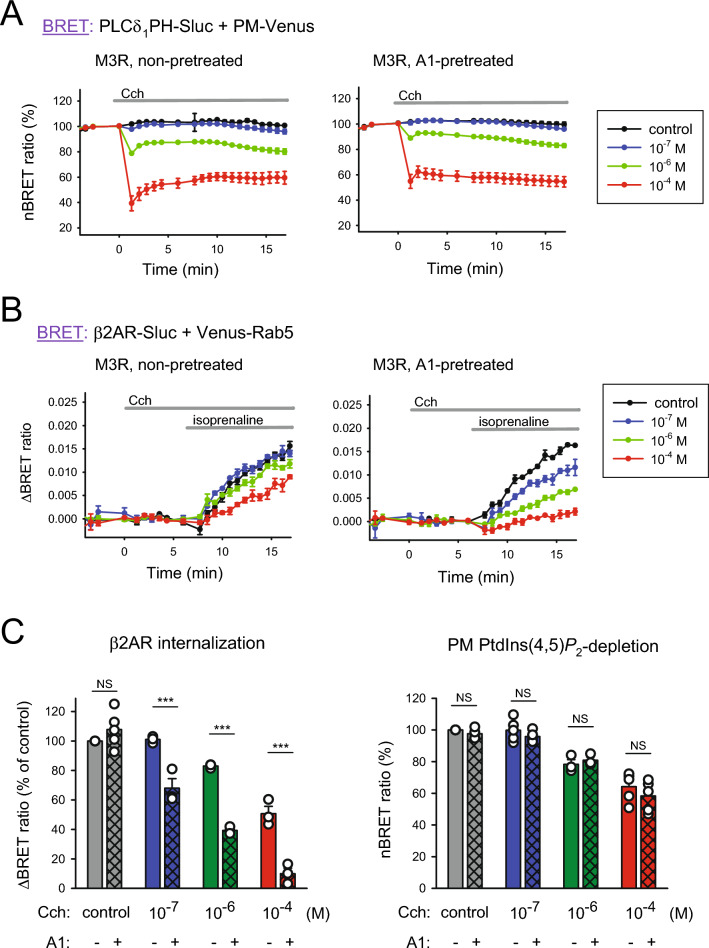
Effect of M3R activation-evoked PM PIP2 depletion and PI4P resynthesis on β2AR internalization. (**A**) PIP2 depletion efficiency was measured by BRET as detailed earlier in Fig. 1B, with the difference that cells expressed type-3 muscarinic acetylcholine receptors (M3R), and lipid depletion was triggered by stimulation with the M3R agonist carbachol (Cch, concentrations as indicated) at time point 0 after pretreatment for 10 min with either vehicle (left graph) or A1 (10 nM, right graph). The signal change was normalized to the last data point before CCh stimulation (100%). Black curves show unstimulated samples. Data show the means ± SEM of 3 independent experiments, each performed in triplicate. (**B**) Internalization of β2AR was measured by BRET between β2AR-Sluc and Venus-Rab5 as detailed in Fig. 2A, with the distinction that M3R was expressed in all cells instead of AT1R-FRB. Cells were pretreated with either vehicle (left panel) or A1 (10 nM, right panel) for 10 min. M3R was stimulated at time point 0 with CCh (concentrations as indicated on the graph) for approximately 5 min, followed by β2AR-Sluc stimulation with isoprenaline (1 μM). The BRET ratio change was calculated by subtracting baselines not stimulated with isoprenaline. Data show the mean ± SEM of 3 independent experiments, each performed in triplicate. (**C**) The bar graph on the left shows the rate of β2AR-Sluc internalization 10–15 min after isoprenaline (1 μM) treatment under the marked circumstances, normalized to the nonpretreated and non-PIP2-depleted sample (clear gray bar). Data are means (± SEM) of the last 5 measurement points of the curves from panel B, using the same color code. The graph on the right shows the rate of PIP2 depletion under the same conditions. Bars show the means (± SEM) of the last 5 measurement points of the curves from panel A, using the same color code. Data were analyzed using two-way ANOVA, which showed a significant difference (*p* < 0.01) between the groups. The Holm‒Sidak post hoc test was used for comparisons within groups, and the results are indicated over the bars (NS—not significant, ***p < 0.001). n = 3.

First, we measured the level of PM PIP2 with the BRET method in control (Fig. 5A left panel) and A1-pretreated (10 nM, 10 min) cells (right panel) using 10^–7^ M (blue traces), 10^–6^ M (green traces) and 10^–4^ M (red traces) carbachol (Cch) stimuli. As shown, activation of M3R with 10^–7^ M Cch causes only a modest if any decrease in PM PIP2, but higher doses of Cch cause a dose-dependent drop. When comparing the control cells and those in which PI4KA was inhibited, we could not detect a substantial difference in the level of PIP2 depletion.

Then, our interest turned again toward the endocytosis of β2AR. The left panel of Fig. 5B shows the BRET ratio changes that we could detect between β2AR-Sluc and Venus-Rab5 upon 1 μM isoprenaline stimulus in control cases (black curves) or after a 5-min pretreatment of cells with 10^–7^ M (blue traces), 10^–6^ M (green traces) or 10^–4^ M (red traces) Cch. The results indicate that only robust PLCβ activation (evoked by 10^–4^ M Cch) and thus remarkable PIP2 depletion could result in a noticeable alteration of β2AR internalization. When experiments were carried out in the presence of A1 (10 nM) (Fig. 5B right panel), we found that even a very low concentration of Cch (10^–7^ M) and thus minor PLCβ activation caused an unequivocal decrease in the rate of β2AR internalization, and the extent of inhibition increased in parallel with the elevating doses of Cch. To make the results easily comparable, we again illustrate our results on a bar scale (Fig. 5C), showing the extent of internalization (left panel) based on the BRET values measured 10 min after the β2AR agonist was added to control (clear bars) and A1-pretreated cells (square grid bars). The changes in PM PIP2 levels are also illustrated for the same time points (right panel). The data clearly show that neither the resting level of PIP2 nor the extent of PLCβ-induced PIP2 depletion was altered significantly by PI4KA inhibition. It also did not affect β2AR endocytosis when PLCβ was not activated, but there was a significant inhibition when M3R and thus PLCβ were stimulated by even the lowest applied Cch concentration. This finding also confirms that PI4KA activation is essential after Gα_q_ stimulus to maintain the hormone-sensitive PIP2 pool, which is indispensable for receptor-mediated endocytosis.

### Testing the effect of PM PIP2 resynthesis on AT1R endocytosis using several endosomal markers

**Figure 6:**
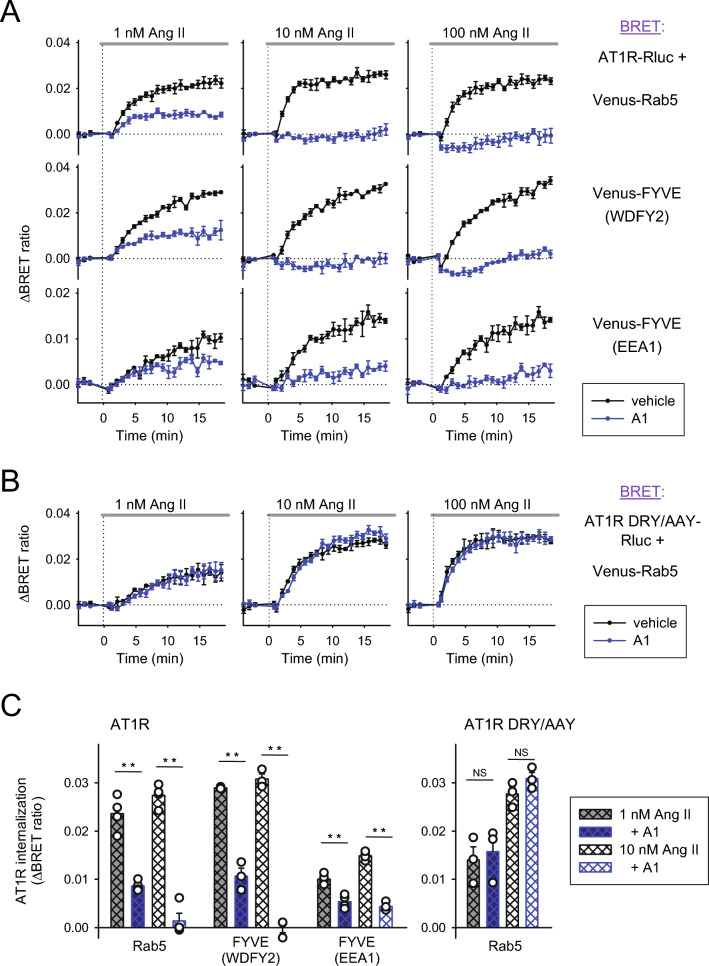
Effect of local PI4P resynthesis on AT1R internalization. (**A**) Internalization of AT1R was measured by BRET in HEK 293T cells between AT1R-Rluc and three different endosomal marker proteins: Venus-Rab5 (upper row), Venus-FYVE (WDFY2) (middle row) and Venus-FYVE (EEA1) (lower row). Cells were pretreated with either vehicle (black curves) or A1 (10 nM, blue curves) for 10 min. AT1R-Rluc was stimulated with Ang II (1 nM, 10 nM and 100 nM for the left, middle and right columns, respectively) at time point 0. The BRET ratio change was calculated against nonstimulated baselines (not shown) for each curve. Data are the mean ± SEM of 3 independent experiments, each performed in triplicate. (**B**) Internalization experiments of the upper row in panel (**A**) were repeated with the G-protein activation deficient mutant of AT1R (DRY/AAY). This time, BRET was measured between AT1R DRY/AAY-Rluc and Venus-Rab5, as detailed in panel (**A**). (**C**) The bar graphs show the rate of AT1R-Rluc internalization 15–20 min after AngII treatment as measured by BRET between AT1R and endosomal markers as indicated. Data are means (± SEM) of the last 5 measurement points of the curves from the left and middle columns of panels (**A**) and (**B**). Data were analyzed using one-way ANOVA for each group separately, which showed a significant difference between samples within each group (*p* < 0.001 for all three groups with wild-type AT1R, *p* = 0.002 for AT1R DRY/AAY). The Holm-Sidak post hoc test was used for pairwise comparisons within groups, and significance is marked between corresponding vehicle- and A1-pretreated samples. **p < 0.01; NS *p* > 0.05; n = 3.

After confirming that PI4KA-mediated resynthesis of PIP2 is essential for β2AR endocytosis when PLCβ is activated, we tried to further simplify our system by testing the endocytosis of the same receptor that induces PIP2 depletion. In this way, we could eliminate the possibility of any direct interaction between the two stimulated receptors which we could not entirely rule out in our previous setups. For these BRET experiments we used the *Renilla* luciferase-tagged version of AT1R, and in addition to Venus-Rab5 we paired it with two additional Venus-tagged endosomal markers: the FYVE domains of the WD repeat and FYVE domain containing 2 (WDFY2) and early endosome antigen 1 (EEA1) proteins. These three proteins have been reported to localize to distinct and only partially overlapping sets of early endosomal vesicles {#47; #46), and by comparing their interaction with AT1R, we could assess whether the receptor is trafficked to different endosomal compartments upon PIP2 depletion. As shown in Fig. 6A, we stimulated AT1R-Rluc with 3 different concentrations of Ang II in vehicle- and A1-pretreated (10 nM, 10 min) HEK 293 T cells (black and blue curves, respectively). Without the inhibitor pretreatment, all 3 endosomal markers displayed a considerable signal increase after stimulation with the lowest Ang II concentration (1 nM, left column, black curves), which became more pronounced by 10 nM Ang II (middle column, black curves) but could not be further enhanced with the highest agonist dose (100 nM, right column, black curves). A1 pretreatment caused a partial inhibition of this signal at a 1 nM agonist concentration (left column, blue curves), which became a total or near-total suppression at both higher doses (middle and right columns, blue curves). We attribute the partial inhibitory effect at the lowest dose to the lower rate of PIP2 depletion induced by receptor activation, as seen previously with M3R-induced lipid degradation (Fig. 5A–C).

To further prove that PLCβ-induced PIP2 depletion plays a crucial role here, we repeated the experiments in the upper row of Fig. 6A with a mutant version of the receptor (AT1R DRY/AAY-Rluc), which is incapable of Gq-protein activation but still internalizes normally upon stimulation {#48; #16; #14}. As shown in Fig. 6B, A1 pretreatment had no effect on the endosomal arrival of the DRY/AAY mutant AT1R after Ang II stimulation, regardless of the agonist dose.

As before, we also summarized our data on bar graphs for clarity and better comparison and subjected these data to statistical analysis (Fig. 6C). The signal amplitude after wild-type AT1R-Rluc stimulation, as well as the rate of A1-induced internalization inhibition, was slightly different for each endosomal marker. However, the effect of A1 pretreatment proved to be significant in all three cases and for both agonist concentrations tested. These data indicate that endosomal arrival of internalized receptors is substantially reduced under these conditions. On the other hand, A1 treatment did not alter the internalization of the Gq activation-deficient AT1R DRY/AAY, indicating once again that PI4KA inhibition alone does not interfere with receptor endocytosis but prevents it when combined with PLCβ-induced PIP2 degradation.

## Discussion

In our previous work, we showed that reducing plasma membrane PIP2 levels significantly inhibits clathrin-mediated endocytosis. In the current work, we were interested in whether this inhibition depends on the mechanism by which PM PIP2 is degraded. Previously, PIP2 depletion was achieved by a rapamycin-evoked translocation of a 5-poshphatase to the FRB-domain that was anchored to the inner surface of the PM by adding the N-terminal targeting sequence of the Lyn protein. This time, instead of this construct, we fused the FRB domain to the C-terminal end of AT1R, allowing both the previously applied rapamycin-evoked and a more physiological, agonist-induced receptor- and consequential PLC activation-based depletion of PM PIP2. By properly adjusting the experimental conditions, approximately 5–6 min after the administration of rapamycin or angiotensin, we were able to achieve almost identical PM PIP2 depletion in both cases as measured by the PLCδ_1_-PH domain-based biosensor. β2AR stimulation by isoprenaline at this time point indicated that regardless of the mechanism by which PIP2 was depleted, β2AR internalization was practically abolished in both cases. Since the addition of Ang II also triggers the endocytosis of AT1R, before concluding that receptor endocytosis does not depend on the mechanism of PM PIP2 depletion, we had to rule out the possibility that the inhibition upon AT1R activation was due to competition between both receptors for the endocytic machinery. The easiest way to do this was to use a previously described internalization-impaired mutant of AT1R, in which 2 threonines and 2 serines in the C-terminal tail of the receptor are replaced by alanins (TSTS/4A)^15^. Surprisingly, when experiments were performed using AT1R-FRB (TSTS/4A), even though PIP2 depletion was slightly larger in response to Ang II than to rapamycin, the process of β2AR internalization was somewhat delayed, but its extent was essentially unaffected. The same result was observed with wild-type AT1R-FRB in cells overexpressing β-arrestin 2, which also indicates that the amount of β-arrestin 2 is a rate-limiting step of internalization. After solving this issue, since agonist-induced PM PIP2 depletion did not diminish receptor internalization, we concluded that the effect on receptor internalization depends on the mechanism of PM PIP2 depletion; moreover, the PM PIP2 level measured by the PLCδ_1_-PH domain-based sensor is of little relevance for this regulation.

The development of protein domain-based inositol lipid biosensors paved the way for a better understanding of the role and importance of these lipids. In the more than twenty years since then, largely thanks to these biosensors, our knowledge of the distribution, quantity changes and regulation of inositol lipids within the cell has increased significantly. However, we have also learned that the lipid binding affinity of these biosensors, their possible interaction with other molecules, and the nonhomogeneous membrane distribution of inositol lipids themselves greatly influence and limit the conclusions to be drawn from experimental results. In the first publication describing a biosensor that allowed the monitoring of PIP2, the title had to be modified to indicate that there was a discrepancy between the absolute mass of PIP2 and the amount of PIP2 detected by the biosensor^13^. The existence of spatially and functionally different PM PIP2 pools is frequently discussed in the literature. We also tried to approach this phenomenon either by changing the PM recruitment of enzymes in the rapamycin-induced system or by changing the PM-targeted Venus by using different PM anchors: L10 for the ordered membrane and S15 for the disordered membrane. Unfortunately, these attempts were not successful^12^, mainly because of the low temporal resolution of the BRET measurement, which allowed the equilibration of the spatially separated pools, even if they existed.

What is the main difference between rapamycin-evoked and agonist-induced PM PIP2 depletion? Based on recent work by the Hammond group, the steady-state level of PM PIP2 can be imagined as a freely and rapidly diffusible pool^29^. Although the PM localization of PLCβ activated by receptor stimulation and the 5ptase recruited to the membrane by rapamycin may show microdomain-dependent differences, their effect on PIP2 degradation can be assumed to be very similar. The steady-state level of PIP2 depends not only on degradation but also on synthesis, and we can expect a significant difference between the two mechanisms. While 5ptase recruitment has no effect on the resynthesis of PM PIP2, it is known that stimulation of many PM receptors, including EGF and several Gq-coupled receptors, activates PI4K and therefore increases the resynthesis of both PI4P and PIP2^12,30^. As shown in Fig. 4, inhibition of resynthesis with A1 only slightly affected the decreased steady-state PIP2 level measured after 5–6 min of stimulation with angiotensin II but completely prevented endocytosis, indicating the importance of locally synthetized PIP. This conclusion was confirmed by visualizing the internalization of β2AR with confocal microscopy and further validated in another system where M3R was used to affect PM PIP2 levels. Like AT1R, M3R is Gq-coupled as well, but it shows no receptor internalization, which eliminates the problem of competition for β-arrestin 2. As summarized in Fig. 5, activation of the receptor with carbachol results in a dose-dependent decrease in measurable PM PIP2 levels, which is not significantly dependent on the presence of a PI4K inhibitor. In contrast, although measurable PM PIP2 depletion evokes a decrease in β2AR endocytosis, this can be further inhibited when the resynthesis of PI4P and PIP2 is abolished. To further simplify the model system, we repeated the internalization measurement using AT1R both as the internalizing receptor and the one responsible for lipid depletion. It was previously published that A1 pretreatment can inhibit AT1R internalization upon its maximal activation^22^; now, we show that the A1 sensitivity of internalization depends on the level of receptor activation and completely disappears when the Gq-activation impaired mutant of AT1R (DRY/AAY) is used. These findings are completely consistent with the results observed in the case of M3R activation-dependent β2AR internalization.

Regarding the importance of steady-state PIP2 levels for receptor endocytosis, our results do not contradict the current consensus. The central role of PIP2 in GPCR-β-arrestin interactions has been confirmed recently in two elegant studies proposing that PIP2 binding may promote an “active-like” conformation for β-arrestins^31^ or even drive their weak, spontaneous PM association, which then facilitates receptor-β-arrestin interactions^32^. In light of these findings, an overall reduction in steady-state PM PIP2 levels might slow the initiation of receptor endocytosis, which could explain the delayed kinetics of our endosomal receptor signals even when the end-point signal amplitude was not significantly reduced (e.g., Fig. 4B, left graph). However, our data highlight that locally resynthesized PIP2, which is not measurable by currently available methods, seems to play a distinct, crucial regulatory role in PM-related functions.

What do we mean by local synthesis of PM phospholipids and how does it relate to the examined receptors? Recently, an increasing amount of data have been accumulating that indicate the formation of protein complexes in certain microdomains of the plasma membrane. In a recent paper from the Baskin lab^33^, the membrane localization of PI4KA was investigated, and they described a palmitoylation code precisely controlling the subdomain-specific localization of the enzyme. In this local environment, changes in phosphoinositide levels may diverge from the overall and experimentally measurable changes in these lipids. Our functional data suggest that the investigated receptors are most likely incorporated into such subdomains, perhaps become parts of enormous molecular complexes that can be regulated by local phospholipid levels. Further development of phosphoinositide sensors to detect lipid levels with increased spatial resolution specifically within these microdomains could be the next step for better understanding PM phosphoinositide metabolism.

## Methods

### Materials

Molecular biology reagents and Lipofectamine 2000 were purchased from Invitrogen. GeneCellin transfection reagent was obtained from BioCellChallenge. Rapamycin was obtained from Selleckchem. Coelenterazine *h* was purchased from Regis Technologies. Unless otherwise stated, all other chemicals and reagents were purchased from Sigma.

### DNA constructs

To prepare the AT1Rs-FRB-mRFP, the sequences coding rat type-1A AT1Rs (WT or TSTS/4A) were inserted using NheI and BamHI into a previously created Clontech pEGFP-N1 vector-based FRB-mRFP construct. The expressed fusion protein has a DPTRSANSGAGAGAGAILSR linker between the receptor and FRB and a YPYDVPDYAPVAT linker (HA-tag) between FRB and mRFP. The BRET-based single-plasmid PM PIP2 sensor (L10-Venus-T2A-PLCδ1PH-Sluc) was described previously^34^. The mRFP-FKBP-5ptase used in pair with AT1Rs-FRB-mRFP to deplete PM PIP2 and the β2AR-Sluc used in pair with Venus-Rab5 to perform the BRET-based measurement of receptor endocytosis were described previously^23^. Other constructs of the rapamycin PM PI4P-depletion system (mRFP-FKBP-Sac1 or mRFP-FKBP-PJ in pair with PM-FRB, which contains the N-terminal targeting sequence of mouse Lck protein) were also described previously^12,27^. β-arrestin 2-mRFP was created by replacing the C-terminal luciferase in a previously described construct^23^ with mRFP using NheI and KpnI. Wild-type human M3 cholinergic receptor (N-terminal 3 × -hemagglutinin tagged) was purchased from S&T cDNA Resource Center. Rluc-tagged rat AT1a receptor and its DRY/AAY mutant were described previously^21^, as well as Venus-FYVE (WDFY2)^23^. Venus-FYVE (EEA1) was generated using the previously described Sluc-FYVE (EEA1) construct^35^, in which Sluc was replaced by Venus. mRFP-tagged AT1R TSTS/4A was generated using the previously described AT1R-Rluc TSTS/4A construct^23^, in which Rluc was replaced by mRFP. All Venus proteins used in this study contain the monomeric mutation (A206K).

### Cell culture

HEK 293 T and HEK 293A cells (ATCC and Thermo Fisher Scientific) were maintained in Dulbecco’s modified Eagle’s medium (DMEM, Lonza 12–604) supplemented with 10% fetal bovine serum, 50 U/ml penicillin and 50 µg/ml streptomycin in a 5% humidified CO_2_ incubator at 37 °C in 10 cm tissue culture plastic dishes.

### Bioluminescence resonance energy transfer measurements

HEK 293T cells were trypsinized and plated on poly-L-lysine-pretreated (0.001%, 1 h) white 96-well plates at a density of 7 × 10^4^ cells/well together with the indicated DNA constructs (0.24–0.3 µg total DNA/well) and the cell transfection reagent (0.5 μl/well Lipofectamine 2000 or 1.5 μl/well GeneCellin) in 150 μl/well Opti-MEM reduced serum medium (Gibco). After 6 h, 100 μl/well DMEM containing serum and antibiotics was added. Measurements were performed 24–27 h after transfection. Before measurements, the medium was replaced with a prewarmed modified Krebs–Ringer buffer (50 μl) (120 mM NaCl, 4.7 mM KCl, 1.2 mM CaCl_2_, 0.7 mM MgSO_4_, 10 mM glucose and 10 mM Na-HEPES, pH 7.4) and incubated for 45 min at 37 °C. Measurements were performed at 37 °C using a Thermo Scientific Varioskan Flash or LUX reader. Each run of the measurement started with the addition of the cell permeable luciferase substrate coelenterazine *h* (40 µl, final concentration of 5 µM), and counts were recorded using 485 and 530 nm emission filters. The detection time was 500 ms (Flash) or 250 ms (LUX) at each wavelength. The indicated reagents were also dissolved in modified Krebs–Ringer buffer and were added manually in 10 µl. For this, plates were unloaded, which resulted in an interruption in the recordings. All measurements were performed in triplicate. BRET ratios were calculated by dividing the 530 nm and 485 nm emission intensities and normalized to the baseline. Because in the case of the intermolecular PIP2 sensors the absolute ratio values depended on the expression, the resting levels were considered as 100%, whereas 0% was determined either by adding ionomycin at the end of the measurement or from the values of those experiments where cytoplasmic Renilla luciferase construct was expressed alone. In those experiments, when the BRET ratio was measured between β2AR-Sluc and Venus-Rab5, the difference in the BRET ratios was calculated between the stimulated (1 μM isoprenaline) and vehicle-treated cells.

### Confocal microscopy

HEK 293A cells at a density of 3 × 10^4^ cells/well were cultured on poly-L-lysine-pretreated (0.001%, 1 h) Ibidi 8-well µ-Slides. After one day, the culture medium was changed to 200 µl transfection solution containing the indicated DNA constructs (0.3 µg total DNA/well) and 0.33 µl/dish Lipofectamine 2000. After 6 h, the transfection solution was changed to 300 µl supplemented DMEM culture medium. Experiments were performed 24–27 h after transfection at 37 °C. Receptor internalization was terminated by fixing the cells in 2% paraformaldehyde (PFA) for 10 min on ice. Confocal images were acquired using a Zeiss LSM710 scanning confocal microscope and a 63x/1.4 oil-immersion objective. Postacquisition picture analysis was performed using Fiji and Photoshop (Adobe) software to expand to the full dynamic range, but only linear changes were allowed.

### Mathematical and statistical analysis

For statistical comparison, unpaired two-tailed t test and one-way or two-way ANOVA with Bonferroni's or Holm-Sidak post hoc tests were applied, as indicated in the figure legends.

### Supplementary Information

Supplementary Figure S1.

## Data Availability

The datasets generated during and/or analyzed during the current study are available from the corresponding author upon reasonable request.
