# Supplementary material for: Effect of hormone-induced plasma membrane phosphatidylinositol 4,5-bisphosphate depletion on receptor endocytosis suggests the importance of local regulation in phosphoinositide signaling
Source: Sci Rep. 2024 Jan 2;14:291. doi: 10.1038/s41598-023-50732-x (PMC10761818; doi:10.1038/s41598-023-50732-x)
Supplement: Supplementary file 1 — Supplementary Figure S1. [file 41598_2023_50732_MOESM1_ESM.pdf]

Effect of hormone-induced plasma membrane phosphatidylinositol 4,5-bisphosphate depletion on receptor endocytosis suggests the importance of local regulation in phosphoinositide signaling

Dániel J. Tóth[1,2,*], József T. Tóth[1,3,*], Amir Damouni[1], László Hunyady[1,4] and Péter Várnai[1,2,#]

[1]Department of Physiology, Faculty of Medicine, Semmelweis University, Budapest, Hungary
[2]ELKH-SE Laboratory of Molecular Physiology Research Group, Eötvös Loránd Research Network, Budapest, Hungary
[3]Department of Anaesthesiology and Intensive Therapy, Faculty of Medicine, Semmelweis University, Budapest, Hungary
[4]Institute of Enzymology, Research Center for Natural Sciences, Centre of Excellence of the Hungarian Academy of Sciences, Budapest, Hungary
*These authors contributed equally.

Running title: Hormone-induced PIP2 depletion and endocytosis
Keywords: PIP2, BRET, Plasma membrane, Endocytosis, GPCR

#Corresponding author:
Péter Várnai, MD, PhD, DSc
Department of Physiology, Faculty of Medicine
Semmelweis University, Budapest
PO Box 2
H-1428 Budapest, Hungary
e-mail: varnai.peter@med.semmelweis-univ.hu

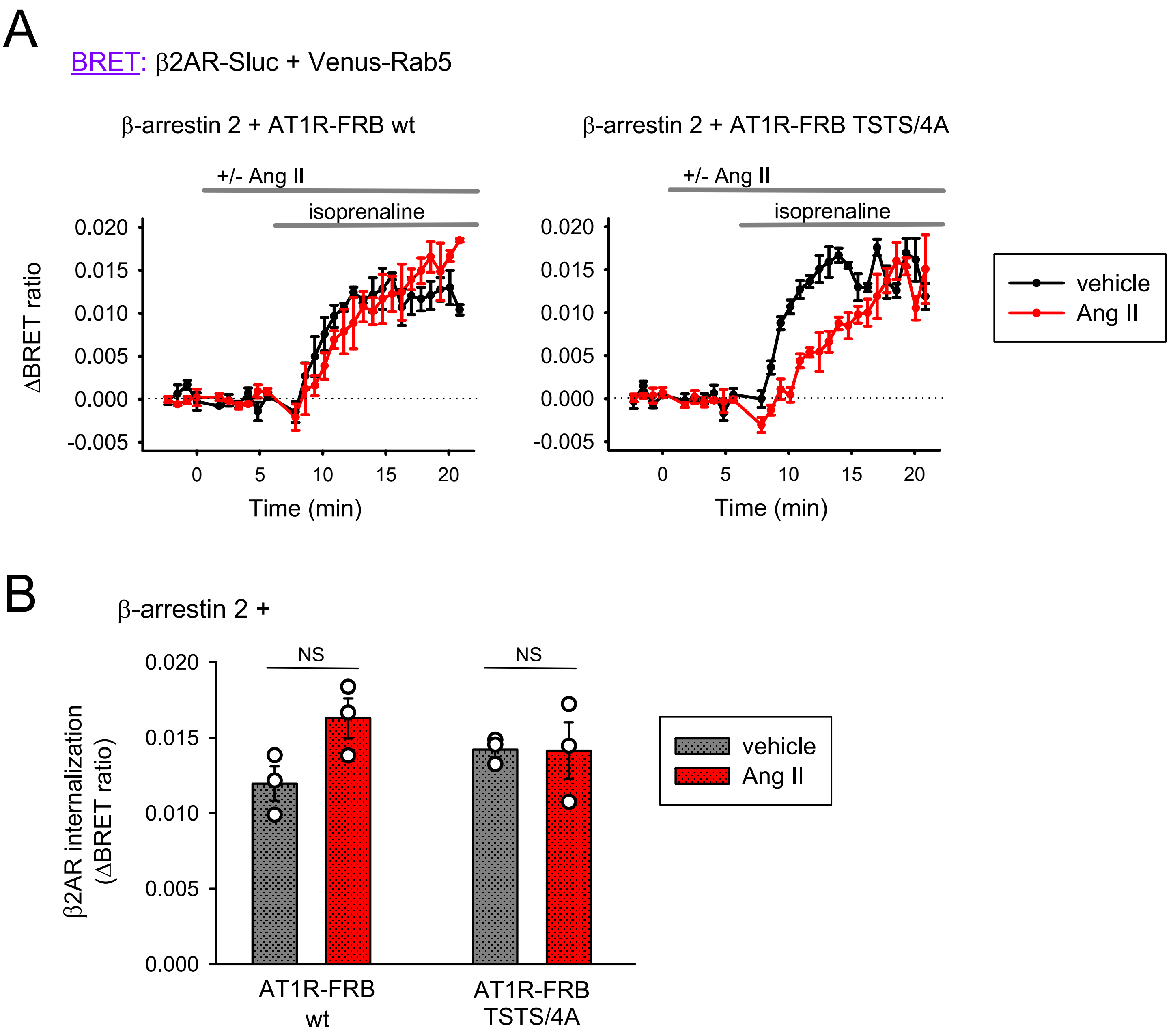

Supplementary Figure 1. The effect of β-arrestin 2 overexpression on β2AR endocytosis after hormone-induced PM PIP2 depletion

(A) Internalization of β2AR was measured by BRET between β2AR-Sluc and Venus-Rab5 as detailed on Fig. 2a, with the distinction that β-arrestin 2 was also expressed in all cells. Cells were treated with vehicle (black curves) or Ang II (100 nM, red curves) for about 5 min before β2AR-Sluc was stimulated with isoprenaline (1 µM). BRET ratio change was calculated by subtracting baselines not stimulated with isoprenaline. Data show mean ± SEM of 3 independent experiments, each performed in triplicate.

(B) Bar graphs show the means (± SEM) of the last 5 measurement points of the curves from panel A. Data were analyzed using two-way ANOVA which showed no significant difference ($p>0.05$) between the groups. n=3

Supplementary Figure 1
